# Supplementary material for: Are carbon emissions trading and green financial instruments synergistic? -Comprehensive quantitative research based on content analysis
Source: PLoS One. 2024 Mar 7;19(3):e0298601. doi: 10.1371/journal.pone.0298601 (PMC10919601; doi:10.1371/journal.pone.0298601)
Supplement: S1 Data — (ZIP) [file pone.0298601.s001.zip › Data/地级市Z_F工作报告.zip!/╔╜╬≈/╤⌠╚¬╩╨/└┤╫╘╣1⁄2╓┌║┼í╛┬φ┐╦╩2╛▌═°í┐_2017.docx]

各位代表：

本届政府任期已满。现在，我代表市人民政府向大会报告工作，请予审议，并请市政协委员和其他列席人员提出意见。

一、过去五年工作回顾

过去的五年，是我市发展史上极不寻常的五年。面对复杂严峻的经济形势，全市上下在省委、省政府和市委的坚强领导下，主动适应、把握、引领经济发展新常态，着力稳增长、促改革、调结构、惠民生、防风险，凝心聚力，顽强拼搏，经济社会各项事业都取得了新成绩。

——五年来，我们综合施策、精准发力，经济在克服困难中稳定增长。面对持续加大的经济下行压力，认真贯彻落实国家、省稳增长的各项政策措施，建立了部门、企业联席会议制度，出台了金融支持实体经济发展意见等政策措施，设立了综合产业扶持基金、企业资金链应急周转保障资金和中小企业发展基金，开展干部入企帮扶，全力支持企业脱困，切实保障工业运行。特别是充分发挥投资对经济增长的关键作用,累计实施各类重点工程项目1201项，完成投资2569亿元，其中省级重点工程投资完成率连续五年进入全省前列。大力开展招商引资，累计到位资金1708亿元，一批具有辐射带动作用的大项目、好项目落户阳泉。五年来，全市地区生产总值年均增长4.8%；全社会固定资产投资年均增长12.6%；社会消费品零售总额年均增长9.8%；城镇、农村常住居民人均可支配收入年均分别增长7.4%和9.6%。

——五年来，我们调优结构、调强动能，转型发展迈出坚实步伐。提出“28字”调产思路，下大力改造提升传统产业，发展壮大新兴产业，着力构建多元支柱产业体系。煤炭“去产能”工作顺利推进，产业集中度大幅提升，22座改造矿井建成投产，原煤入洗率由40.5%提高到66.7%。电力产业发展取得重大突破，西上庄2×66万千瓦、鑫磊2×35万千瓦低热值煤发电项目推进顺利，裕光2×100万千瓦发电项目获得核准；我市获批采煤沉陷区国家先进技术光伏示范基地，一期100万千瓦项目正在加快推进。新兴产业发展加快，百度云计算中心稳定运行并不断扩大应用领域，阳煤乙二醇一期20万吨项目即将投产，中兴环能纳米洋葱碳项目实现量产，快递人均发生量领跑全省，旅游总收入年均增长22.5%。产业结构不断优化，服务业占GDP比重上升至50.3%。特别是大力推进开发区改革创新发展，扩区调规、平台建设加快推进，转型发展迈出坚实步伐。

——五年来，我们统筹城乡、建管并重，新型城镇化进程不断加快。覆盖城乡的重大基础设施建设力度空前。阳五、太阳、西环、阳左高速相继建成通车，形成了100公里环城高速圈，高速公路密度达到6.27公里/100平方公里，位居全省第一。人民群众期盼的阳大铁路开工建设、推进顺利，累计完成投资24.8亿元。汽车客运南站主体完工。城市饮用水水质改善、娘子关二期供水、污水处理二期三大水务工程竣工投用。市政建设迈出新步伐。实施道路畅通工程，泉西路、保晋路、广阳路、华盛桥改扩建、平坦立交桥改续建、政府广场和沃尔玛地下通道等建成投用，漾泉大道、平阳街、宁波北路加快推进。供水普及率、燃气普及率、集中供热率、垃圾无害化处理率分别达到100%、91.1%、90.2%和83.3%。5个省级重点镇建设累计完成投资37.9亿元。城镇化率达到66.68%，居全省第二。环境综合整治扎实有效。持续改善城市人居环境，市容市貌整治成效明显，21个城中村改造项目推进较快。特别是去年以来，我们排除各种阻力，推进南大街、义平路两条样板街（路）建设，共拆除建筑物259处，面积近12万平方米；整饰楼体129处，建设绿地39处，完成“三线”入地、公交港湾建设、路面和人行道维修等整治工程。同时，农村人居环境改善“四大工程”完成投资33.2亿元，建成8个省级美丽宜居示范村。智慧阳泉建设成效明显。在全省率先实现主要公共场所无线互联网免费全覆盖，智慧阳泉数据中心建成并投入使用，成功入选“宽带中国”示范城市。

——五年来，我们强农惠农、精准帮扶，“三农”工作成绩显著。各级财政对“三农”投入累计达到91.6亿元。积极实施产业富民工程，形成了以畜牧、核桃、蔬菜为主导，小杂粮、中药材、食用菌、薯类、养蜂、休闲观光竞相发展的特色农业格局。严格落实耕地保护责任，粮食产量连续多年稳定在2.2亿公斤以上。新发展农民专业合作社1552个、“一村一品”专业村296个。大寨核桃露等7家企业年销售收入过亿元，九州香公司富硒小米销往20多个省市，康泰来公司食用菌远销美国、韩国，全市农产品加工龙头企业销售收入由2011年的9亿元增长到28亿元。2016年精准脱贫8843人、退出贫困村45个，脱贫攻坚首战告捷。

——五年来，我们深化改革、锐意创新，发展活力进一步彰显。国有企业改革不断深化，南煤集团成功重组，国有商贸服务企业全部实现民营化改制，水泵阀门“退城进园”、蔬菜市场搬迁等项目进展顺利。财税改革稳步推进，财政预算绩效管理走在全省前列。国有林场主体改革顺利完成，全省排名第一。盂县农信社在全省率先改制为农商行。全省首家专门服务“三农”的盂县汇民村镇银行挂牌成立。平定县入选全省“普惠金融试验区”。天元绿环、云泉岩土公司在“新三板”挂牌上市。建成科技孵化器、众创空间等双创孵化平台14家，市科技孵化器升级为国家级孵化器，我市成为全省首家创新型城市试点市。先后争取到国家老工业区搬迁改造、独立工矿区改造搬迁政策，获批国家中小城市改革试点、国家产城融合示范区。

——五年来，我们保障民生、繁荣文化，人民群众得到更多实惠。在财政十分困难的情况下，民生支出占一般公共预算支出的比重稳定在75%左右。城镇累计新增就业12万人，棚户区改造、保障性住房、农村危房改造等指标完成省定任务；城镇登记失业率、居民消费价格指数涨幅，均低于省控目标；城乡低保标准、农村五保供养标准排全省前列。在全省率先实现60岁以上农民基础养老金发放全覆盖。合并实施城镇居民养老保险和新型农村养老保险制度。新农合参保率达到99%以上，城乡居民大病保险实现全覆盖。在全省率先实现国家义务教育发展基本均衡县（区）全覆盖。高考录取率稳居全省前列。太原理工大学阳泉学院成功“升本”，更名为“山西工程技术学院”。启动职业技术学院新校区建设。覆盖城乡的现代公共文化服务体系基本建成, 荣获全国文化体制改革先进市称号。刘慈欣作品《三体》获世界科幻大会雨果奖。成功举办全国蹦床冠军赛，阳泉籍运动员王智伟、武杨、涂潇在国际国内重大赛事上摘金夺银。安全生产工作连续多年走在全省前列，各类事故起数和死亡人数逐年“双下降”，2016年煤矿百万吨死亡率为零，被确定为全国遏制重特大事故试点城市。

——五年来，我们节能减排、治理环境，生态文明建设成效突出。组织实施重点节能项目70个，形成年节约83.2万吨标准煤能力，万元GDP能耗完成省定任务。加大环境治理力度，特别是去年以来，面对严峻的环保形势，大力开展“铁腕治污行动”，坚决打好“七大攻坚战”，全力实施“三个清零行动”和“六项整治”工作，集中开展“土小”企业专项整治，共取缔“土小”竖窑1150支，根治了困扰我市多年的顽疾，受到省通报表扬。加大水污染防治力度，启动实行“河长制”。经过不懈努力，环境质量持续改善，扭转了一段时间环保工作被动局面。累计完成各类营造林59万亩，建成区绿化率达到41.4％，森林覆盖率达到28.5%，获得“全国绿化模范城市”荣誉称号。

——五年来，我们依法行政、强化效能，政府执行力和公信力明显提升。严格执行人大及其常委会决议、决定，积极支持人民政协参政议政，共办理人大代表议案和建议925件、政协委员提案1851件。认真落实全市政府系统管党治党政治责任，共查处违纪违法案件357件506人。全面推进依法行政，强化行政监察和审计监督，制定了人事调动、预算外资金分配、工程项目招投标管理等制度办法。政务公开工作排全省前列。进一步简政放权，累计取消行政审批事项231项、下放74项、调整16项、承接206项。完成市、县政府机构改革和公车改革。积极创新政府服务手段，阳泉“随手拍”运行以来，注册用户超过5万，入驻单位351家，网友问政办结率达到90%以上。认真开展党的群众路线教育实践活动、“三严三实”专题教育和“两学一做”学习教育，严格执行中央八项规定和国务院“约法三章”，坚决反对“四风”，“三公”经费占公共财政支出比重由2012年的2.54%下降到0.64%。

与此同时，加强国防动员和后备力量建设，深入开展双拥工作，大力发展妇女儿童、老龄、残疾人和红十字会等事业，民族宗教、侨务等工作受到国家表彰。外事、气象、地震、人防、档案、史志等工作都取得了新的成绩。

过去五年，是我市发展史上最为困难、最为曲折的时期。面对复杂严峻的外部经济形势和内部结构性矛盾双重压力，我们既重视在供给侧方向发力，加大结构性改革力度，又着力扩大有效需求，推动投资、消费较快增长；既在传统产业改造上做文章，全力稳增长、促发展，又在新兴产业培植上下功夫，努力打基础、利长远；既注重内部挖潜，勒紧裤带过“紧日子”，又注重眼睛向外，积极做好招商引资、向上争取等工作；既着眼于现代化城市发展目标，不断加快城市建设、生态环保等硬环境建设，又借鉴发达地区经验，致力于打造服务最优、效率最高、诚信最佳的经济发展软环境。五年来，经过全市上下的共同努力，尽管一些经济指标完成不尽如人意，但社会事业和民生领域工作始终走在全省前列，一些多年想干未能干的大工程、大项目相继上马或陆续建成，发展成果最大限度惠及全市人民。去年，全市经济增长在经历连续几年下行的形势下，呈现出低位企稳、逐步向好的发展态势，各项先行指标持续走强，全社会用电量、铁路货运量连续多月在全省处于领先位置；主要经济指标逐步回升，地区生产总值比上年加快2.3个百分点，规上工业增加值比上年加快6.2个百分点。可以说，我们在压力下赢得了主动，在困难中收获了成就，经济运行中趋势性、标志性积极因素不断增多，为今后发展奠定了良好基础。这一局面的形成，是省委、省政府和市委坚强领导、统揽全局的结果，是市人大、市政协大力支持、有效监督的结果，是全市广大干部群众同心同德、团结拼搏的结果。在此，我代表市人民政府，向全市人民，向各位人大代表、政协委员，向各民主党派、工商联和无党派人士，向驻地部队、武警官兵、公安干警和中央、省属驻地单位，向所有关心支持参与阳泉改革发展的同志们、朋友们，表示衷心的感谢和崇高的敬意！

各位代表，尽管我们取得了一定的成绩，但要清醒地认识到，阳泉经济走出低谷，走向转型升级、创新发展的新路，还需要继续爬坡过坎、不懈奋斗。多年积累的产业结构单一、发展方式粗放、体制机制僵化、创新创造不足等深层次矛盾和问题远未从根本上解决；国有企业活力不强，民营经济贡献不大，实体经济运行困难，财政收支矛盾突出，经济增长缺乏大项目、好项目支撑，短期内很难实现跳跃式增长；城市建设欠账较多，生态治理、环境保护与人民群众的期盼还有较大差距；一些干部担当意识不强，干事劲头不足，工作中存在不敢为、不会为的现象，招商引资办法不多，抓落实的力度不大等问题依然存在。这些问题已经严重阻碍了我市经济社会的持续健康发展，必须下大力加以解决！

二、今后五年政府工作的目标任务侯卫东官场笔记

今后五年是我市深化供给侧结构性改革和转型综改试验区建设的关键时期。做好今后五年的工作, 必须把我市放在国际、国内发展的大背景下来审视和谋划。一方面，必须清醒地看到，世界经济持续低迷，国内经济发展进入新常态，增速换挡、转型升级、动力转换是一个长期渐进的过程。传统产业产能过剩、传统产品市场需求不旺，对我市主要依靠传统要素驱动、资源型产业主导的经济结构带来重大挑战，也对就业、财政、居民增收等带来巨大压力。另一方面，综合来看，今后五年阳泉发展总体上处于可以大有作为的重要战略机遇期。从国内来看，中央坚持新发展理念，把科技创新、自主创新摆在国家发展的战略地位，百度、阿里巴巴、华为等一批世界领先企业活力四射，新一轮科技革命带来的新技术、新产业、新业态蓬勃发展，全国各地正在积极谋划新一轮产业项目，抢占发展制高点，必将为我市依靠科技创新、培育战略性新兴产业提供历史性机遇。从全省来看，以省第十一次党代会为标志，我省实现了战略重点转移，主动转型、创新转型、深度转型、全面转型正成为发展主旋律，特别是省委、省政府围绕深化转型综改、加快国资国企改革、推动开发区二次创新创业等，正在出台和即将出台一批重大改革举措，这些必将为我市资源型经济转型创造政策机遇。从我市来看，加快发展的积极因素不断累积，投资环境和发展条件正在改善。特别是市第十二次党代会提出了“四大定位”，市委十二届二次全会暨经济工作会议，进一步完善了发展思路，全市上下凝聚起转型升级、创新驱动的磅礴力量。为此，我们务必要把困难估计得更充分一些，把工作准备得更周密一些，把前途看得更光明一些，进一步增强忧患意识、机遇意识、创新意识、争先意识，坚定信心，迎难而上，全力以赴做好今后五年的工作。

今后五年的重点工作目标任务是：

（一）坚持创新驱动，在建设“转型发展先行市”中走出阳泉新路。抓创新就是抓发展，谋创新就是谋未来。要坚定不移地实施创新驱动战略，将综改试验区建设与供给侧结构性改革相结合，突出科技创新和开发区建设的引领作用，进一步完善“28字”调产思路，加快转型升级步伐。未来五年，“三去一降一补”任务取得实质性进展，化解煤炭过剩产能1285万吨，建成千万千瓦级现代综合能源基地，传统产业在工业中的占比控制在60%以内，煤炭行业增加值占比下降到50%左右；信息技术、文化旅游、新材料、新能源等战略性新兴产业占比达到18%以上；服务业增加值占GDP一半以上，民营经济份额达到50%左右，研发经费占GDP比重达到2.5%。未来五至十年，开发区改革创新发展大步推进，建成1个国家级经济技术开发区、3个省级经济技术开发区，每个开发区面积达到50平方公里左右。开发区成为全市转型综改主战场，成为集聚要素最多、创新活力最强、发展环境最优的经济增长平台，率先在全省走出资源型城市转型升级的新路！

（二）坚持经营推动，在建设“晋东区域中心城”中彰显阳泉气魄。在城市建设中树立“经营”理念，以市场化手段建设和管理城市，多渠道筹措建设资金。坚持规划先行、分步实施，建设“生态、智慧、宜业、宜居”的现代化城市。建成国家卫生城市、省级文明城市。积极创建全国智慧城市。继续加大旧城改造力度，重点改造提升交通、管网等基础设施，完善城市综合服务功能，切实改善人居环境；持续推进生态新城建设，按照新城总体规划，加快推进基础设施和公共服务设施建设，高标准、高质量打造新城起步区。抓好大县城和中心镇建设，提高城镇对人口和产业的承载力。把阳泉建设成为重要的商贸物流、医疗卫生、文化旅游、教育科技、金融信息中心，打造内涵更加丰富的区域中心城市！

（三）坚持区域联动，在建设“城乡统筹示范区”中再创阳泉佳绩。新型城镇化是扩大有效需求的潜力所在。依托我市城镇化水平较高的优势，加快推进规划编制、基础设施、公共服务、产业布局、制度安排“五个一体化”。合理安排市域县域城镇建设、农田保护、产业聚集、村落分布、生态涵养等空间布局，推动水电路气等基础设施共建共享，促进教育、医疗、文化、社保等基本公共服务均衡配置。到2020年，高质量高标准完成农村整体脱贫任务，全面建成小康社会；到2021年，全市城镇化率达到70%以上，城镇新增就业10万人，城乡发展一体化和区域协调发展格局基本形成，城乡居民共享改革发展成果！

（四）坚持外向带动，在建设“山西向东开放桥头堡”中展示阳泉形象。开放是一个地区形象与活力的展示，也是富民强市的必由之路。要“对表”中央要求，对标先进发达地区，对接国际惯例，全面优化发展环境，以海纳百川的胸怀，有效吸引国内外资金、技术、人才、管理等有形无形资源为我所用，创造先进生产力。进一步完善招商引资政策，下功夫引进一批科技含量高、发展前景好、引领带动强的大项目、好项目，进一步扩大经济总量。充分发挥区位优势，积极参与京津冀协同发展、环渤海合作发展和“一带一路”建设的分工协作，加强与长三角、珠三角等沿海地区项目合作，建设更加开放、更具活力、更有优势的外向型经济新体系！

各位代表，今后五年的工作有挑战，更有机遇，我们有决心、有能力担负起人民赋予的神圣使命，坚定发展信心，保持战略定力，不断克服前进道路上的各种困难和挑战，勇敢走出一条符合阳泉实际的创新驱动、转型升级之路，创造更加美好的明天！

三、2017年工作安排

2017年是实施“十三五”规划的重要一年,也是推进供给侧结构性改革的深化之年。政府工作的总体要求是：全面贯彻党的十八大和十八届三中、四中、五中、六中全会精神，深入贯彻习近平总书记系列重要讲话精神和治国理政新理念新思想新战略，统筹推进“五位一体”总体布局，协调推进“四个全面”战略布局，根据省第十一次党代会和省委全会暨经济工作会议以及市第十二次党代会部署，按照省委“一个指引、两手硬”重大思路和要求，牢固树立和落实新发展理念，坚持稳中求进工作总基调，坚持以提高发展质量和效益为中心，坚持深化供给侧结构性改革与深化转型综改试验区建设有机结合，坚持以开展“全面落实年”活动为抓手，全面实施创新驱动、转型升级战略，全力促进经济稳步向好和社会和谐稳定，不断塑造美好形象，逐步实现振兴崛起，以优异成绩迎接党的十九大胜利召开。

主要预期指标是：地区生产总值增长5%；规上工业增加值增长3%；社会消费品零售总额增长7%；一般公共预算收入降幅控制在5%以内，力争持平；城镇常住居民人均可支配收入增长6%，农村常住居民人均可支配收入增长6%以上；城镇新增就业2万人，登记失业率控制在4.2%以内；居民消费价格涨幅控制在3%左右。

约束性指标是：资源节约方面，万元地区生产总值能耗下降3.2%，万元地区生产总值二氧化碳排放量下降3.9%，万元地区生产总值用水量下降2.5%。环境质量方面，包括细颗粒物（PM2.5）浓度下降比例，城市空气质量优良天数比例，达到或好于Ⅲ类水体比例，劣V类水体比例，二氧化硫、化学需氧量、氨氮、氮氧化物减排幅度，完成省下达任务。民生改善方面，农村贫困人口脱贫8261人、贫困村退出54个。基本建成保障房9000套，改造农村危房710户。

确定这些目标，充分考虑了当前与长远、速度与转型、内部与外部等因素，体现了稳中求进的总基调。需要指出的是，固定资产投资增长目标根据新的统计口径研究确定，更加注重优化投资结构，提高精准性、有效性，把重点放在扩大产业投资、促进民间投资上。财政支出坚持“紧日子、保基本、调结构、保战略”，发挥好“四两拨千斤”的作用。约束性指标既是转型的标尺，也是基本的民生要求，必须不折不扣完成。

重点抓好以下几方面工作：

（一）以开发区建设统领转型升级。

推动开发区改革创新发展。把开发区建设作为转型综改的战略突破口，认真落实全省重大战略部署，突出抓好整合、改制、扩区、调规，确保我市新一轮开发区建设开好局、起好步、见成效。整合阳泉东区工业园，加快推进阳泉经济技术开发区扩区；以平定龙川工业园为主体，设立平定经济技术开发区；以盂县牛村煤电化工业园为主体，设立盂县经济技术开发区；以郊区苇泊高新装备制造园为主体，设立阳泉荫营经济技术开发区。开发区要按照“七通一平”标准加快基础设施建设，年内重点打造2平方公里路网、1平方公里建设用地，至少落地1个亿元以上主导产业项目。坚持“五规合一”，科学编制开发区建设总体规划和产业发展规划。强化开发区经济职能，社会事务原则上交由所在地政府承担。深化开发区“三化三制”改革，理顺开发区管理体制、运行机制和各种利益关系，切实向开发区简政放权，激发开发区发展活力。构建电子政务管理服务体系，实行“一站式”政务服务大厅和“五证合一”登记制度，切实提高开发区办事效率。制订出台开发区改革创新工作考核办法，明确工作要求和标准，奖优罚劣，形成开发区建设相互赶超、争先进位的良好局面。

加快产业转型升级步伐。产业是经济的基础，项目是产业发展的重要抓手，进一步完善“28字”调产思路，突出信息，加强物流，年内重点抓好九大产业100个项目，完成投资180亿元。

煤炭产业。继续推进煤炭“去产能”工作，年内关闭阳煤五林井和盂县万和兴2座煤矿，退出产能150万吨。坚持淘汰落后产能与发展先进产能相结合，推动平定卫东、盂县路家村、辰通3座煤矿建成投产，推进阳煤一矿、程庄等9座煤矿先进产能认定，先进产能占比达到50%。全年煤炭产量控制在5600万吨左右，原煤入洗率保持在70%以上，入选率稳定在90%以上。瓦斯抽采量达到19.4亿立方米，利用率达到40%以上。

电力产业。继续推进西上庄2×66万千瓦、鑫磊2×35万千瓦低热值煤发电等项目建设，推动裕光2×100万千瓦项目尽快开工。加快采煤沉陷区光伏示范基地一期100万千瓦项目建设进度，确保6月底前并网发电。积极做好盂县、平定、郊区风电项目前期工作。

冶金产业。按照“稳定氧化铝，配套电解铝，加快发展铝精深加工”的思路，大力支持阳煤兆丰与山东信发等企业合作，积极发展铝合金、铝型材、铝铸件等系列产品。做好晋东50万吨铝循环产业园区建设前期工作，推动煤电铝全产业链一体化发展，全力打造晋东铝工业基地。

煤化工产业。围绕“肥、醇、烯”产品路径，构建新型煤化工和特色精细化工两大产业体系，推动煤化工产业向技术高端和价值链高端提升。加快阳煤乙二醇二期30万吨项目及聚酯等终端产品项目前期工作，培育煤炭—乙二醇—聚酯纤维产业链。

装备制造业。以高端化、专业化、智能化为目标，引导企业发展智能制造、绿色制造和高端装备制造。加快推进神盾智能公司光电数控设备设计制造、华骏机械公司年产5000台减速机等项目。

新材料产业。以精深加工和提升附加值为方向，积极发展高档磁性材料、高端陶瓷、高强度陶粒砂等新材料产业。重点推进山西贝特瑞新能源3万吨高端人造石墨等项目建设。整合重组现有耐火产业和企业，形成几个科技含量高、市场前景好的拳头产品和企业集团。　　信息产业。以百度云计算中心为龙头，以云谷创业科技园为载体，以智慧城市建设为基础，延伸云计算上下游产业链条，发展以软件、硬件、电子商务、物联网为主要内容的信息产业。加快推动百度云计算中心二期投运，支持百度公司建设山西区域性云计算大数据应用交易中心和“百度创新中心”,争取建成全省大数据物联网中心。坚持把发展大数据产业与“智慧阳泉”建设相结合，构建以城市大数据发展为核心的智慧城市发展框架。

现代物流业。充分利用我市承东启西的区位优势，围绕能源矿产品、大宗工业品、生活日用品、特色农产品等，加快现代物流园区、配送中心和大型市场建设，打造晋东区域物流中心。重点要依托优质无烟煤资源，发挥好晋东煤炭交易中心作用，提升晋东联晟物流园和华佳、乱流、东关等发煤站发运规模，有效吸纳陕、蒙及周边地区煤炭资源，按照用户需求提供定制煤炭产品，打造华北具有影响力的煤炭物流基地。

文化旅游业。把文化旅游业作为战略性支柱产业加以培育。做好全域旅游总体规划和重点景区总体规划。加快景区体制改革，理顺所有权、管理权和经营权关系，成立旅游发展委员会。以娘子关、大汖温泉为重点推动精品景区建设。拓宽融资渠道，引进大企业、大集团参股或管理，推进旅游景区市场化运营。加强市场监管，着力打造“安、顺、诚、特、需、愉”的软环境。以京津冀晋为重点，加大“二日游”“三日游”客源市场开发力度，突出发展乡村休闲游、红色革命游、生态风光游，打造特色小镇，增强阳泉景区的知名度。大力培育新型文化市场主体，做大做强刻花瓷、煤雕、砂器等本土特色文化产业。

与此同时，围绕消费促进经济增长，培育发展新兴服务业态，推动服务业转型升级。巩固提升我市电子商务发展态势，积极发展农村电子商务主体，培育向日葵信息技术、红金云网络科技等一批有实力的本土电商企业，促进本土名优特产品网上销售。适应人民群众日益增长的多样化、个性化、信息化消费需求，大力发展教育、体育、健康、休闲、养老等幸福产业，改造提升商贸、餐饮、住宿、家政等传统服务业，推动消费结构优化升级。此外，多措并举稳定房地产消费，完成房地产“去库存”任务。

全力抓好招商引资。只有大招商才有大发展。要围绕开发区建设招商选商，按照开发区既定的主导产业特点确定招商主攻方向，着力引进具有辐射带动作用的大项目、好项目。要围绕产业转型招商选商，抢抓京津冀协同发展、太原都市圈建设等机遇，重点在铝工业、煤化工、文化旅游、信息产业等行业领域，实施精准招商、全产业链招商、集群化招商。要创新招商手段，强化领导招商、乡情招商、“展会+小分队”招商、专业委托招商、商会协会招商等措施，多渠道、全方位做好宣传与项目推介，认真开展“晋商晋才回乡创业创新”工程。要完善招商引资考核办法，重点考核合同履约率、资金到位率、项目开工率、投产达效率，特别是新兴产业占比率。鼓励干部在招商引资中算大账、算长远账、算发展账，敢为客商让利、敢为客商优惠、敢为客商便捷，敢于牺牲局部利益和眼前利益去谋求长远发展、赢得全局主动。确保全年完成项目签约680亿元，外来投资到位300亿元。

加大服务企业力度。总结干部入企服务工作经验，建立服务企业的常态化机制，对重点企业、困难企业施行“一企一策”，最大限度解决企业的困难和问题。坚持与阳煤集团的联席会议制度，搞好协调服务，促进企地共赢。认真贯彻落实各项减税降费政策，切实减轻企业负担，降低企业运行成本，提高企业盈利能力。

各位代表，加快转型升级, 必须从抓载体、抓项目开始。我们就是要以供给侧结构性改革为主线，总体抓平台,部门抓产业，县区抓项目，突出转型，做强实体，优化服务，不断推动产业项目进园区、产业结构多元化，使我市在未来经济发展中赢得主动！

（二）积极推进重点领域改革创新。

深化国企国资改革。当前我市国企国资改革已进入新的历史关口。我们要紧紧围绕“市场化取向、竞争力目标、专业化重组、股份制改造、现代化管理、科学化监管”的重大要求，坚定改、主动改、抓紧改、彻底改，建立真正意义上的现代企业制度。推进股权多元化改革，支持民间资本通过多种方式参与国企改革，逐步实现市属商贸流通企业国有资本退出。依法对“僵尸”企业实施破产关闭，积极盘活闲置资产。进一步完善国有资产管理体制，稳步推进国有企业整合，实施专业化重组、板块化经营。完成水泵、阀门、蔬菜市场“退城进园、搬迁改制”工作。强化沟通衔接，全力推动晋东化工厂搬迁改造迈出实质性步伐。按时完成党政机关与所办企业脱钩改革工作。加快推进厂办大集体改革，解决企业办社会问题。尽快出台集体企业改革办法。

大力发展民营经济。民营经济是转型发展的中坚力量，必须下大力、出实招，全力补齐这块结构性短板，确保年内民营经济增加值占比提高3个百分点。强化对民营企业的服务。认真落实国家、省关于支持民营经济发展的各项政策，保障民营企业平等获得要素支撑、公平参与市场竞争、同等受到法律保护。充分发挥市中小企业公共服务综合平台作用，想方设法为民营企业提供土地、道路、用电、用气等基础条件以及人才、技术、第三方服务等必要条件。扶持民营企业做大做强。坚持“非禁即入”原则，引导民间资本更多地投向电力、旅游、物流、大数据等转型项目以及公共服务、基础设施、城市管理、环境整治等领域。积极探索以联合投资基金方式整合民间资本，推动民营企业通过产业规划和园区承载，形成产业集群。大力推进中小微企业成长工程，新创办市场主体1500家，培育“小升规”企业4家以上。鼓励民营企业创新体制机制。引导民营企业主动适应市场环境，逐步改变“家族式、作坊式”的传统经营模式，通过资本市场运作进行股份制改造，建立现代企业制度，不断提高市场竞争力。关心民营企业家成长。把民营企业家当成宝贵财富，高看一眼，厚待一分，政治上给待遇，工作上给便利，环境上给优惠，着力构建“亲、清”政商关系，让民营企业家放心、放手、放胆工作。

加快金融改革创新。深化地方金融机构改革，加快推进平定、郊区农信社改制。大力支持金融机构“去杠杆”，引导金融机构帮助企业置换债务，有效降低企业负债率。引导企业利用专项建设基金、应急周转保障资金、信用增进、再担保等方式，多渠道降低融资成本。大力推动资本市场发展，推广项目建设PPP融资模式。做大做强现有政策性融资担保公司，充分发挥城投公司政府投融资平台作用，扩大融资规模。优化金融资源配置，引导金融资本、社会资本投资我市重点发展的产业和领域。准确把握国家和省投资重点，抓紧储备、包装一批既符合上级产业政策、又符合我市实际的大项目、好项目，争取更多国省资金投资我市。优化小微企业金融服务，发展农村普惠金融，完善科技、文化、绿色金融服务体系。同时，加强和改进金融监管，严厉打击金融犯罪，确保地方金融稳定。

积极实施科技创新。着力培育创新主体。重点在高端装备制造、新能源、新材料、信息技术等领域培育一批高新技术企业、创新型企业和民营科技企业，着力构建企业为主体、产学研合作相结合的技术创新体系，建立省级以上产业技术创新联盟1家，市级以上工程技术研究中心、重点实验室5家。加快科技成果转化。在煤层气抽采和综合利用、煤机装备制造等领域实施一批重大科技专项和重点科技项目，不断提升产业创新能力。推进科技孵化体系和人才队伍建设。以市高新技术创业园为龙头，完善全市创新创业孵化体系，实现“双创”平台扩容提质。加快三和伟业、乐融众创等双创基地建设，培育省级众创空间2家、市级2家。健全完善人才政策体系，培养用好本土人才，积极引进高端人才。

各位代表，改革创新是经济发展的持久动力。我们就是要把改革创新摆在发展全局的核心位置，努力在重点领域和关键环节的改革创新上取得突破，为全市转型发展注入强大的活力。

（三）加快推进新型城镇化步伐。

推进生态新城建设。加大基础设施建设力度。重点抓好漾泉大道一期复工，推进漾泉大道二期续建，实施漾泉大道一期、二期节点改造工程；开工建设宁波北路、平阳街西延、洪城北路北延等工程，推进新城支路路网建设。尽快开工建设综合交通客运枢纽工程。加快公共服务设施建设。启动实施中心医院、多功能文化综合体、中央森林公园一期等项目，基本完成职业技术学院新校区建设。继续推进桃坡、余积粮沟、三泉、大西庄、魏家峪等保障房项目。创新投融资机制。设立我市改善城市人居环境PPP投资引导基金，通过争取国家投资、吸引民间资本、经营城市资产等方式，多渠道筹集新城建设资金。

强化城市综合服务功能。继续抓好一批路网工程。开工建设中环快速路，这条路以在建的漾泉大道、平坦路、现状义白路、义平路局部为基本框架，南接平定，西连矿区，北穿生态新城，东进开发东区，全长26.1公里，建成后将极大缓解城市交通压力，促进市区扩容提质，对于阳泉转型发展、统筹城乡发展具有里程碑意义。继续加快推进阳大铁路工程，重点抓好沿途车站建设。实施国道207、307改线工程。做好太旧高速公路南连接线前期工作。推进汽车客运南站正式运营。同时，用好“两区”政策，加快实施城市排水管网建设改造、城镇周边煤矿瓦斯综合利用、城区老工业区供热、供气、供水管网设施更新改造等工程。大力推进海绵城市建设。加快实施城市地下综合管廊工程。积极推进城区老旧片区改造。新开工建设10个停车场，逐步缓解我市停车难问题。全面运行数字城管系统，建设县区数字城管二级平台。

加大城乡环境综合整治力度。继续完善南大街、义平路环境综合整治工程。围绕解决“脏、乱、差”问题，重点抓好主要河道、主次干道、背街小巷等区域的综合整治，坚决治理乱扔乱倒、乱搭乱建、乱停乱放等城市“顽疾”。推进生活垃圾中转站、餐厨垃圾和有机废物综合处理、建筑垃圾资源利用一体化工厂等项目建设。启动实施李家庄河与甄家庄河河道治理工程。完成棚户区改造任务。大力推进城中村改造,新启动15个村7760套住房建设。同时，继续推进农村人居环境改善“四大工程”，加快农村饮水安全、移民搬迁、污水垃圾处理等基础设施建设，抓好3个县区9个乡镇采煤沉陷区治理搬迁安置工作，启动110户地质灾害避让搬迁。深化农村“两治三改”。争创2-3个省级美丽宜居示范村。

各位代表，今年，我市将全面启动国家卫生城市创建，力争2019年城市区申报国家卫生城市；2018年至少有一个农业县区申报国家卫生县城，每个县区至少有一个乡镇申报国家卫生乡镇。全市上下要紧紧围绕现代化城市建设目标，以攻坚克难的勇气，坚持不懈的努力，使城市面貌“洗心革面”，城市建设“脱胎换骨”！

（四）加大生态环保力度。

强力推进环境污染综合整治。今年，我市被列入“2+26”通道城市，我们要坚决打好大气、水、土壤污染防治攻坚战，确保全市环境质量持续改善。大气污染防治方面，重点深化四项措施：控煤，建成区内全部淘汰20蒸吨及以下燃煤锅炉，实现高污染燃料禁燃全覆盖，完成省下达的以气代煤、以电代煤任务；降尘，加强道路扬尘治理，提高道路机扫率，加快推进裸露土地硬化、绿化工作；治矸，抓住全省生态恢复试点建设政策机遇，加大治理力度，逐步根治矸山污染；管车，从严落实“三个严禁”，完成省下达的黄标车、老旧车淘汰任务，加快推进纯电动公交车、出租车更新替代工作。建立重污染天气应急预警长效机制，坚决服从国家和省统一调度，严格落实应急减排措施，做到响应及时、令行禁止。水污染治理方面，对境内37条重点河流全面实行“河长制”，落实属地责任，健全长效机制，切实加强河流管理保护工作；加快推进重点镇污水处理设施新建工程，提高污水管网覆盖率和污水处理率；大力推进娘子关水源地保护工作。土壤污染防治方面，深入开展土壤环境质量调查，加强土壤污染源监管，严防土壤污染。同时，深入开展“铁腕治污行动”，对环境质量恶化、重大环境问题整改不到位、部门履职不到位和监管责任不到位的，坚决严肃问责。

推进能源资源集约节约利用。全面推行工业、建筑、交通、公共机构等行业领域能效对标，强化重点用能企业节能管理，继续实施一批节能技术改造项目，推进土地、矿产集约节约高效利用。

持续加快生态建设。以增绿增质增效为重点，加快实施退耕还林、贫困山区造林、森林管护等工程，高标准完成各类营造林10万亩。实施好国家水土保持重点建设项目，完成水土流失治理14万亩。

各位代表，建设美丽阳泉，是全市人民的共同心愿。我们要始终坚持绿色发展理念，以改善环境质量为重点，更加注重标本兼治，努力把阳泉建设成为生态宜居的美好家园。

（五）统筹做好“三农”和脱贫攻坚工作。

扎实推进农业供给侧结构性改革。适应农产品消费升级需要，主打“特色”、“优质”两张牌，大力发展现代农业、功能农业。调结构，今年要调减玉米种植面积3万亩，同时，扩大谷类、薯类种植面积，继续抓好核桃、优质杂粮、食用菌、中药材、养蜂等特色产业发展。建基地，立足比较优势，优化区域布局，打造一批特色农产品生产基地，加快“三带三区”集聚形成。扬龙头，充分发挥龙头带基地联农户作用，重点推进康泰来、万和油脂等续建项目，全市农产品加工企业销售收入力争达到30亿元，增长7%。强基础，继续推进农村安全生命防护工程和旅游公路建设。开工建设乌河水库，积极做好龙华河调水、檀山沟水库建设等前期工作。优服务，加强新型职业农民培育，实施农业科技创新行动计划，建立完善科技、人才、金融、流通、政策五位一体服务体系。推改革，深化农村综合改革，稳步推进农村集体产权制度改革，开展清产核资，明晰农村集体产权归属，赋予农民更充分的财产权利。完善农村土地所有权、承包权、经营权分置机制，发展适度规模经营，增加农民财产性收入。

大力推进脱贫攻坚。坚持把脱贫攻坚工作摆在更加突出的位置，完善“五个一批”脱贫路径，突出产业扶贫、易地搬迁扶贫、金融扶贫等工作重点，确保完成年度脱贫任务。通过发展种植、养殖、旅游等产业脱贫4125人；通过易地搬迁脱贫1071人；通过退耕还林、荒山造林、森林管护等，生态脱贫1065人；通过对贫困家庭普通高中贫困生免除学杂费、贫困家庭子女考入中职以上院校给予补助等方式，教育脱贫1000人；通过建立健全新型社会保障体系，社会兜底脱贫1000人。强化对脱贫攻坚的评估考核，坚决防止数字脱贫、弄虚作假，确保真扶贫、扶真贫、真脱贫。

（六）切实保障和改善民生。

着力稳定和扩大就业。大力实施高校毕业生就业创业促进计划。做好“去产能”职工安置工作。统筹做好农村转移劳动力、城镇失业人员、“4050”人员、退役军人等群体就业工作。实施职业培训全覆盖计划，提升劳动者就业创业和职业转换能力。

完善社会保障体系。推动机关事业单位养老保险制度改革入轨运行。实现城乡居民基本医保制度并轨，提升医疗保障水平。提高城乡低保标准和特困人员救助供养标准，推动农村低保和扶贫开发两项制度有效衔接。实施残疾人精准康复服务行动。做好全民参保登记试点工作。

促进各类教育协调发展。发展有质量的普惠性学前教育。巩固义务教育均衡发展成果，合理配置教育资源。做好中考改革试点工作，优化发展高中阶段教育，着力构建现代职业教育体系。认真落实特殊教育提升计划。全面深化中小学教师职称制度改革，进一步改善和优化教师队伍结构，全面提高教育教学质量。

提升公共文化服务水平。健全政府购买公共文化服务机制，实施文化惠民演出、广播影视有效覆盖和公益电影进社区工程，广泛开展群众性文化活动。繁荣文艺创作，讲好阳泉故事，开展纪念建市70周年系列文艺创作展演活动。大力推广“全民阅读”。加大文物和非物质文化遗产保护力度。推进“乡村文化记忆”工程。

大力发展卫生体育事业。巩固县级公立医院改革成果，深化城市公立医院综合改革，全部取消药品加成。实施药品采购“两票制”改革，推进城乡药品配送一体化。加强医疗质量监管，不断深化平安医院建设。大力推进分级诊疗和家庭医生签约服务工作。加大农村医疗人才培养力度。深化计生服务管理改革，促进人口均衡发展。加快推进健康阳泉建设，广泛开展全民健身，进一步提高竞技体育水平。

支持国防和军队建设，加强国防动员、国防教育和后备力量建设，做好优抚安置和人防工作，推进军民融合深度发展，争创“全国双拥模范城”。发展妇女儿童、老龄、残疾人和红十字会等事业。做好统计、地震、民族宗教、外事、侨务、档案、史志等工作。

各位代表，我们要牢固树立以人民为中心的发展思想，努力破解民生难题，积极回应民生关切，切实解决民生需求，让人民群众有更多的获得感和幸福感！

（七）下大力抓好安全稳定工作。

毫不放松抓好安全生产。牢固树立安全发展理念，坚持“党政同责、一岗双责、企业（单位）主责，失职追责”，压实各级政府领导责任、行业部门监管责任。强化企业主体责任，切实做到安全责任、安全投入、安全培训、基础管理、应急救援“五到位”。健全完善安全监管体制机制，切实加强安监队伍建设。深入推进遏制重特大事故试点城市工作，全面构建安全风险管控和隐患排查治理双重预防性工作体系，加快实施阳泉智慧安监等保护生命重点工程，提升安全生产保障水平。继续深化煤矿、非煤矿山、道路交通、危险化学品、建筑施工、油气管道、特种设备、人员密集场所等重点行业领域专项整治。全面落实新修订的《山西省安全生产条例》，引深“打非治违”专项行动，对非法违法行为零容忍、严查处，推动安全生产形势根本好转。

全力维护社会稳定。推进社区“网格化”管理，构建城乡社区、社会组织、社会工作联动机制。依法有序抓好信访维稳工作，及时妥善处理群众诉求。健全应急管理和防灾减灾体系，进一步提高突发事件处置能力。加强食品药品监管，加快建立重要产品追溯体系。深化平安阳泉建设，狠抓社会治安综合治理，努力营造和谐稳定的社会环境。

各位代表，在转型综改的关键时期，安全稳定工作显得尤为重要。我们一定要以铁的担当、铁的手腕、铁的心肠、铁的办法，坚决守住“三条底线”，绝不让安全稳定问题影响干扰转型发展大局！

（八）加强政府自身建设。

建设廉洁政府。严格落实全面从严治党主体责任，严格执行党内政治生活准则，深入推进政府系统党风廉政建设和反腐败斗争。政府系统干部必须切实增强“四个意识”，坚决把纪律和规矩挺在前面，要求别人做到的自己带头做到，要求别人不做的自己坚决不做。全面落实中央八项规定和国务院“约法三章”，牢固树立“过紧日子”思想，严控“三公”经费支出。坚决查处隐形变异“四风”问题，努力营造风清气正、干事创业的政务环境。

建设效能政府。深化行政审批制度改革，严格落实“两集中、两到位”，进一步减少审批事项和审批环节。推进“互联网+政务服务”，依托“智慧阳泉”整合现有网络资源，加快网上服务平台建设，最大限度方便群众办事创业。扎实推进“五证合一、一照一码”改革。依托阳泉“随手拍”建设“12345”市民服务热线，进一步提升为民服务水平。深入开展“全面落实年”活动，切实整治“以文件落实文件、以会议落实会议”等问题。认真执行“13710”工作制度，实行清单管理，强化政务督查，确保各项工作落到实处。

建设法治政府。我们的权力是党和人民赋予的，法定职责必须为，法无授权不可为。严格执行重大行政决策法定程序，实行合法性审查、终身责任追究及责任倒查机制。对公共资金、国有资产、国有资源和领导干部履行经济责任实行审计全覆盖。加强对各类公共资源交易特别是重大项目招投标的监管，坚决杜绝“明招暗不招”、招标“走形式”的歪风。加快推进市县两级市场监管领域综合行政执法体制改革，全面推行“双随机、一公开”。依法接受人大及其常委会的监督，自觉接受政协民主监督，主动接受社会和舆论监督，让权力在阳光下运行，使政府工作经得起法律的审查和历史的检验。

建设担当政府。权力就是责任，责任就要担当。政府系统领导干部一定要义无反顾地肩负起时代的重任，始终保持夙兴夜寐、奋发有为的精神状态，把全部心思用在干事创业上，把满腔热情倾注在为民造福上。认真贯彻市委《鼓励改革创新激励干事创业容错纠错实施办法》，为敢担当的干部担当，为敢负责的干部负责，鼓励和引导干部在克服困难中解决问题，在化解矛盾中推动工作，充分调动各方面力量攻坚克难，不断开创转型发展新局面、再铸阳泉新辉煌。

各位代表，转型综改、创新驱动、全面小康、振兴崛起，是时代赋予我们的历史使命。让我们更加紧密地团结在以习近平同志为核心的党中央周围，在省委、省政府和市委的坚强领导下，忠诚担当，锐意进取，顽强拼搏，狠抓落实，为把我市建设成为“山西向东开放桥头堡、晋东区域中心城、转型发展先行市、城乡统筹示范区”作出新的更大贡献！
